# Accreditation Procedure for *Trichinella* spp. Detection in Slaughterhouses: The Experience of an Internal Laboratory in Italy

**DOI:** 10.3390/foods8060195

**Published:** 2019-06-06

**Authors:** Maria Schirone, Pierina Visciano, Alberto Maria Aldo Olivastri, Maria Paola Sgalippa, Antonello Paparella

**Affiliations:** 1 Faculty of Bioscience and Technology for Food, Agriculture and Environment, University of Teramo, 64100 Teramo, Italy; mschirone@unite.it (M.S.); apaparella@unite.it (A.P.); 2 Azienda Sanitaria Unica Regionale Marche, 63100 Ascoli Piceno, Italy; albertoolivastri@libero.it; 3 FreeLance Veterinary, 63100 Ascoli Piceno, Italy; mariapaola.sgal@libero.it

**Keywords:** *Trichinella* spp., slaughterhouse, accreditation, proficiency testing

## Abstract

Trichinellosis is a severe foodborne zoonotic disease due to the consumption of undercooked meat containing *Trichinella* spp. larvae. According to Commission Regulation (EU) No 1375/2015, domestic pigs, farmed wild boar, and horses must be tested for the presence of the parasite in the muscles as part of post-mortem examination. In this study, the accreditation procedure and the maintenance of the certificate for internal laboratory attached to a slaughterhouse are described. The main advantages of such accreditation are represented by the possibility to obtain fast results in order to process carcasses quickly, whereas the difficulties for the technician are linked to performing proficiency testing and following training courses. This program can be considered particularly useful for surveillance and food safety purposes.

## 1. Introduction

Trichinellosis is one of the most important foodborne parasitic diseases caused by the consumption of raw or undercooked meat of swine, horses, and wild animals infected by the nematode larvae of *Trichinella* spp. [1]. The genus *Trichinella* involves nine species and three genotypes, distinguished in encapsulated (*Trichinella spiralis*, *Trichinella nativa*, *Trichinella britovi*, *Trichinella murrelli*, *Trichinella* T6, *Trichinella nelsoni*, *Trichinella* T8, *Trichinella* T9, and *Trichinella patagoniensis*), transmissible only to mammals, and non-encapsulated (*Trichinella pseudospiralis*, *Trichinella papuae*, and *Trichinella zimbabwensis*), infective to mammals, birds, and reptiles. The first three cited species as well as *T*. *pseudospiralis* show a high pathogenicity to humans. With regards to their distribution, *T*. *spiralis* is reported worldwide and can have a domestic and sylvatic life cycle. Also *T*. *nativa* and *T*. *britovi* have been described in Europe [2,3]. Turiac et al. [4] reported that *Trichinella* spp. were detected in 354 animals from 1985 to 2016 in Italy, with percentages of 97.5 for *T*. *britovi*, 2.2 for *T*. *pseudospiralis*, and 0.3 for *T*. *spiralis*. 

*Trichinella* spp. recognize a single host in which two generations of the parasite can occur but it does not involve a free-living stage [5,6]. The life cycle consists of three phases: intestinal, migrant, and muscular [7]. In the muscular phase, the larvae can survive from about 1–2 to 10–15 years in hosts because they form a nurse cell–parasite complex into myocytes after de-differentiation and re-differentiation of muscle cells [8,9]. 

The symptoms of trichinellosis in humans can be different on the basis of the phase of the life cycle of the parasite. The disease recognizes five clinical forms: severe, moderately severe, benign, abortive, and asymptomatic [10]. During the intestinal acute phase, diarrhea and abdominal pain are the most common signs preceding fever and myalgia. Migrating larvae cause immunological and metabolic disturbances, with eosinophilia and release of histamine, serotonin, and prostaglandins [11]. Other symptoms of the acute stage are pyrexia, periorbital or facial oedema, and myalgia. Major complications of trichinellosis can be cardiovascular (i.e., myocarditis and tachycardia), neurological (meningitis or encephalopathy), respiratory (dyspnea, pneumonia, and obstructive bronchitis); death from trichinellosis is rare [10]. In Europe, trichinellosis cases in humans were 224 in 2017, with a notification rate of 0.03 cases per 100,000 populations representing an increase of 50% compared with 2016 [12]. 

The domestic pigs may ingest the parasite by feeding non-cooked scraps or offal from slaughtering. The transmission to humans is generally linked to the consumption of uncooked or undercooked meat from pigs or hunted wild animals ingesting infected rodents and wildlife. During the period 2004–2014, Badagliacca et al. [13] reported 91 wild mammals resulting positive, namely, wolf, red fox, wild boar, stone marten, pine marten, and wildcat. 

The monitoring of the presence of *Trichinella* spp. in animal species susceptible to infection has been implemented since 2006 according to Commission Regulation (EC) No 2075/2005 [14], which allowed risk-based *Trichinella* spp. testing of domestic pigs with derogation for those raised in farms under negligible risk [15]. This Regulation has been repealed by Commission Regulation (EU) No 1375/2015 [16], laying down rules for the sampling of carcasses of domestic swine, horses, wild boar, and other farmed and wild animal species susceptible to *Trichinella* spp. infestation. It also provides for reference and equivalent methods for the detection of parasite larvae in samples of carcasses to be analyzed in a laboratory designated by the competent authority, as reported by Regulation (EC) No 882/2004 [17]. These tests are generally performed by the Istituto Zooprofilattico Sperimentale (IZS) located in different Italian regions, even if also other laboratories may conduct the analysis for official controls as long as they are accredited in accordance to European standards. Such accreditation results from a conformity assessment aiming at verifying whether specific processes, systems, personnel, or organizations comply with specific requirements. Nowadays, the global accreditation system is formed by the International Laboratory Accreditation Cooperation (ILAC), including 120 accreditation bodies across the globe with the purpose to promote the coordination of the accreditation activities [18]. In Italy, the only appointed Italian Accreditation Authority is ACCREDIA since December 2009 in accordance with Regulation (EC) No 765/2008 [19]. It evaluates the technical competence of operators and the credibility of the attestations they release [20]. The aim of this study is the description of the accreditation procedure followed by the internal laboratory attached to an abattoir located in Marche region (Central Italy) for *Trichinella* spp. detection in swine carcasses after the slaughtering process. To our knowledge, this laboratory is one of the only 16 national accredited laboratories placed inside slaughterhouses. The requirements for the maintenance of such accreditation are also reported.

## 2. Materials and Methods 

### 2.1. Preliminary Information

**Figure 1:**
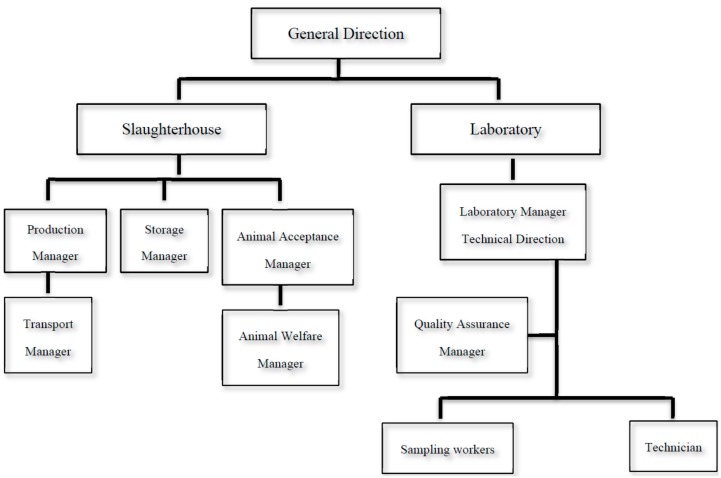
Organization chart reported in the Quality Assurance Manual.

The accreditation procedure described in the present study was assessed in accordance with EN International Organization for Standardization/International Electrotechnical Commission (ISO/IEC) 17025/2005 on “General requirements for the competence of testing and calibration laboratories” except for sampling made by the competent authority and analytical technique following the official method [16]. The internal laboratory analyzed carcasses of domestic swine coming from holdings not officially recognized as applying controlled housing conditions, as well as carcasses of horses, wild boar, and other farmed and wild animal species susceptible to *Trichinella* spp. infestations. The organization chart is reported in Figure 1. By way of derogation from Article 3, paragraph 3 of Commission Regulation (EU) No 1375/2015 [16], carcasses and meat of domestic swine may be exempt from *Trichinella* spp. examination, as reported also by a national document of the Ministry of Health [21], provided that no autochthonous *Trichinella* spp. infestations have been detected in the last three years. Moreover, another national document of the Ministry of Health [22] specifies that the monitoring of *Trichinella* spp. regarding 10% of carcasses is not necessary anymore on the basis of the epidemiological Italian status but remains mandatory only for carcasses of breeding sows and boars. 

The EN ISO/IEC 17025/2005 permits laboratories (public or private, managed by government, industry, or other organizations) to demonstrate that they work expertly and generate valid results that can be accepted by other countries without further analysis. It has been recently revised by EN ISO/IEC 17025/2017, and ILAC in consultation with ISO agreed that a three-year period from the date of publication should be granted to laboratories needing to transition their processes to this new version. During such transition period, both ISO/IEC 17025/2005 and ISO/IEC 17025/2017 could be considered equally valid and applicable [23]. 

### 2.2. Preparation of the Quality Assurance Manual 

The preparation of a Quality Assurance Manual (QAM) was the first step for the accreditation. It can be considered the reference manual for personnel working in the slaughterhouse, animal owners as customers of the abattoir, and ACCREDIA. It defines the quality policy and organization of the company, the procedures, the responsibilities, as well as the methods of performance. In particular, the quality policy, prepared by the Technical Direction of the Laboratory, involved the evaluation of customer’s requests, data obtained, and prospective skills. It was firstly described to the personnel during a meeting and then exposed on a bulletin board. In this context, a Quality Assurance Manager was appointed to check the achievement of such objectives and establish operating procedures and implementation times. The General Direction ensured the availability of adequate resources to obtain the defined skills and verified the whole Quality Assurance System (QAS) whenever a modification of one procedure was required. 

### 2.3. First Accreditation 

The QAM was evaluated by ACCREDIA, and subsequently two auditors visited the laboratory and carefully observed the trial carried out by the internal personnel. After their approval, they conferred an accreditation certificate with a validity for four years. 

### 2.4. Maintenance of the Accreditation Certificate

The inspection from ACCREDIA, as described before, was repeated every four years in order to obtain the accreditation renewal, whereas a surveillance assessment was performed each year to verify that the accredited laboratory continued to meet the requirements throughout the validity period of the accreditation certificate. In addition, a proficiency testing according to Marucci et al. [24] was made once a year on meatballs from female mice artificially infected with *T. spiralis* muscle larvae by the European Union Reference Laboratory for Parasites (EURLP, i.e., the Istituto Superiore di Sanità in Rome, Italy). In particular, the proficiency samples could be used to demonstrate performance at an adequate level of sensitivity and, in such occasion, they were spiked with a low number of larvae (3–5), whereas a higher number of larvae could be suitable for training and troubleshooting purposes. The results of the proficiency testing were good when all added larvae were found and acceptable when at least one larva was identified from each spiked sample. The test failed when no larvae was recovered, and in such case, the analyst competence had to be investigated by reviewing data records and/or retesting [25]. The failure of the proficiency testing could cause the suspension of accreditation until a new compliant result was obtained. 

The operators working in the accredited laboratory required one spiked sample to the EURLP twice a year only for a training purpose, but these results did not affect the accreditation validity. 

### 2.5. Reference Method of Trichinella spp. Detection 

According to Commission Regulation (EU) No. 1375/2015 [16], the magnetic stirrer method for pooled sample digestion was applied. Briefly, 100 g of pooled samples taken from a pillar of the diaphragm of swine carcasses was chopped in a blender containing water, pepsin, and hydrochloric acid. The digestion fluid was stirred until the meat particles disappeared and then poured through a sieve into a sedimentation funnel for 30 min. A portion (40 mL) of the digestion fluid was run off into a measuring cylinder for 10 min, and then 30 mL of supernatant was withdrawn. The remaining 10 mL of sediment was poured into a larval counting basin or Petri dish and examined by trichinoscope or stereo-microscope at a 15 to 20× magnification. 

## 3. Results and Discussion

The results of the present study show the accreditation procedure of the internal laboratory attached to a slaughterhouse in 2012 for the first time and its maintenance up to the current year. The description of the whole process is reported in Table 1, while Table 2 shows QAM, consisting of 19 procedures. Such laboratory was accredited for the analysis of carcasses of swine and other species susceptible to *Trichinella* spp. infection obtained in the slaughterhouse, with an extension also for the examination of carcasses of large wild game and pigs slaughtered at farm level. In order to maintain such accreditation, the technician should: (i) Participate in a training course (at least 8 h once a year), (ii) Pass an annual inter-laboratory test, and (iii) Practice with infected meatball samples every six months. The QAM was revised in four occasions, three of which were due to non-compliances revealed by ACCREDIA, such as the lack of the calibration certificate of a specific instrument, the duplication of some equipment to work faster, or the failure to complete an acceptance report for pigs slaughtered at home. The last revision was made in 2016 after the repealing of Commission Regulation (EC) No 2075/2005 [14] by Commission Regulation (EU) No 1375/2015 [16]. 

The accreditation procedure described in this study involved some difficulties for the technician working in the laboratory, such as the structural features required by ACCREDIA and the training courses to be followed in order to maintain the accredited status. Such courses were rarely organized by IZS, even if a minimum number was not mandatory according to ACCREDIA auditors, and therefore the laboratory could consider performing this task.

Another complication was related to the potential failing of the proficiency testing for both the maintenance of accreditation and the training of personnel. In the first case, besides a detailed report of this failure to provide to ACCREDIA auditors, other three spiked meatballs should be purchased from EURLP by the technician, with an expensive charge for the laboratory. For a training purpose, just one spiked sample was considered sufficient. In the present study, the results of the proficiency testing were always acceptable because 2–3 out of four larvae (spiked samples) were detected. This quantitative evaluation was based on the Z-score established by EURLP. 

**Figure 2:**
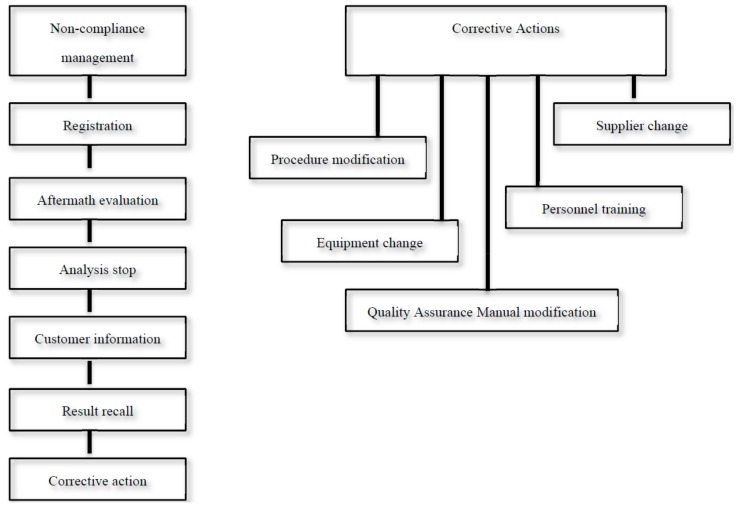
Management of non-compliance and corrective actions (procedures P7 and P8 in Table 2).

The management of non-compliance and corrective actions (Procedures P7 and P8 reported in Table 2) made by the QAM is shown in Figure 2. Such non-compliance could be observed both into the logistic cycle of the laboratory (i.e., sampling, transport, storage, analysis, and result communication) and into the consideration of prescriptive standards reported in QAM. 

The advantages of the accreditation of the internal laboratory are represented by the opportunity to get results quickly (2–3 h after slaughtering), whereas if the analysis was made by the official laboratory (i.e., IZS) the results would be delivered after many hours or even the day after the slaughtering. For this reason, the customer owner of slaughtered animals prefers to pay the analysis made by the internal laboratory in order to obtain fast results. Moreover, because of the extension of accreditation also for the analysis of swine carcasses slaughtered at farm level, as well as large wild game, their meat could be manufactured and processed after a short time from slaughtering. Indeed, when the analyses of pigs slaughtered at farm level were made by IZS, the results were provided with a large delay because the competent authority collected the samples only twice a week and delivered them late.

Active *Trichinella* spp. control is essential for ensuring food safety and facilitating international trade. Additionally, for pork carcass safety assurance, considering *Trichinella* spp. risk assessment, the effective control of this hazard depends on preventive measures and controls continuously applied on farm and at the slaughterhouse in an integrated way [26]. The European Legislation requires that carcasses of animal susceptible to such parasite must be examined before they can be intended for human consumption. The possibility of doing such analyses inside a slaughterhouse is useful for customers as well as for the competent authority in guaranteeing the safety of the slaughtered carcasses. Periodic reviews of the entire accreditation procedure must be conducted and planned at least every five years in order to certify the reliability of the results, even if the experience has shown that it represents a complex and laborious process. 

## Figures and Tables

**Table 1:** Description of the activities for the accreditation.

Process	Responsible	Input	Output	Activities
Customer management	QAM	Sample identification	Sample to be analyzed	Draft acceptance sheet
Supplying	TD	Stock management, calibration plan	Products and services	Selection and control of suppliers and products
Sampling	QAM	Competent authority	Laboratory	Sampling from carcasses and identification
Analysis	TD	Sample delivery	Test report	Application of analytical method
Result communication	TD	Test report	Customer and/or competent authority delivery	Registration of delivery and original copy
Resource management	TD	Maintenance, setting and operator qualification plan	Setting, training, proficiency testing, and quality control	Ordinary and special maintenance, setting of equipment, personal training
Revision	GD	Documents	Review report and improvement actions	Process performance analysis

Legend: QAM = Quality Assurance Manager, TD = Technical Direction, GD = General Direction.

**Table 2:** List and description of procedures of the Quality Assurance Manual.

Number	Title	Scope and Application
P1	Protection of confidential information	The laboratory guarantees the confidentiality of customer data
P2	Management of documentation of Quality Assurance System	The external or internal documents are identified, registered, and listed in a specific archive
P3	Definition of the contract	The customers accept and sign the proposal of the contract with the Laboratory
P4	Selection of suppliers	The Laboratory selects a specific list for qualified suppliers of reagents, instruments, and equipment and periodically updates it
P5	Check of reagents and equipment	The Laboratory verifies the correct status of equipment and expiry date and the suitability for use of reagents (e.g., pepsine) for the analysis
P6	Resolution of customers’ complaints	The customers’ complaints are registered in a database and analyzed in order to be solved by corrective actions
P7	Management of non-compliance	If the Laboratory verifies a non-compliance, it prepares a document named “Risk Assessment” with corrective actions to be applied
P8	Management of corrective and preventive actions	The Laboratory evaluates the cause of non-compliance and implements corrective/preventive actions in order to avoid the recurring of such event
P9	Management of audit	The audits are conducted annually aiming at verifying if the Quality Assurance System is compliant with ISO/IEC 17025:2005
P10	Revision by General Direction	The General Direction makes an annual revision of the Quality Assurance System generally after the visit by ACCREDIA
P11	Training of personnel	The Technical Direction guarantees the training of personnel and confirms its effectiveness
P12	Monitoring and maintenance of environmental conditions	The procedures of sanitation are applied daily after the analysis and more accurately each month
P13	Validation of analysis	The validation involves only the good practice of both equipment and technician because the applied method is already officially recognized
P14	Control of data	The Technical Direction examines and signs the test report
P15	Management of equipment	The Laboratory tests the equipment before the analysis in order to check its suitability
P16	Management and setting of instruments	The Laboratory performs the setting of instruments when required
P17	Sampling	The sampling is carried out by qualified personnel under the supervision of the competent authority
P18	Handling and storage of sample	The Laboratory controls the integrity, temperature, and quantity of samples at their arrival
P19	Quality Assurance	The Laboratory assures the quality of the analysis by using spiked meatballs as a reference certified material and participating in inter-laboratory tests

## References

[1] Dimzas D., Diakou A., Koutras C., Gómez-Morales M.A., Psalla D., Keryttopoulos P., Deligianni D., Kontotasios K., Pozio E. (2019). Human trichinellosis caused by *Trichinella britovi* in Greece, and literature review. J. Helminthol..

[2] Rostami A., Gamble H.R., Dupouy-Camet J., Khazan H., Bruschi F. (2017). Meat sources of infection for outbreaks of human trichinellosis. Food Microbiol..

[3] Gómez-Morales M.A., Ludovisi A., Amati M., Cherchi S., Tonanzi D., Pozio E. (2018). Differentiation of *Trichinella* species (*Trichinella spiralis*/*Trichinella britovi* versus *Trichinella pseudospiralis*) using western blot. Parasite Vectors.

[4] Turiac I.A., Cappelli M.G., Olivieri R., Angelillis R., Martinelli D., Prato R., Fortunato F. (2017). Trichinellosis outbreak due to wild boar meat consumption in southern Italy. Parasite Vectors.

[5] Pozio E. (2016). Adaptation of *Trichinella* spp. for survival in cold climates. Food Waterborne Parasitol..

[6] Zarlenga D., Wang Z., Mitreva M. (2016). *Trichinella spiralis*: Adaptation and parasitism. Vet. Parasitol..

[7] Saracino M.P., Calcagno M.A., Beauche E.B., Garnier A., Vila C.C., Granchetti H., Taus M.R., Venturiello S.M. (2016). *Trichinella spiralis* infection and transplacental passage in human pregnancy. Vet. Parasitol..

[8] Xu J., Yang F., Yang D.Q., Jiang P., Liu R.D., Zhang X., Cui J., Wang Z.Q. (2018). Molecular characterization of *Trichinella spiralis* galectin and its participation in larval invasion of host’s intestinal epithelial cells. Vet. Res..

[9] Park M.K., Kang Y.J., Jo J.O., Baek K.W., Yu H.S., Choi Y.H., Cha H.J., Ock M.S. (2018). Effect of muscle strength by *Trichinella spiralis* infection during chronic phase. Int. J. Med. Sci..

[10] Dupouy-Camet J., Kociecka W., Bruschi F., Bolas-Fernandez F., Pozio E. (2002). Opinion on the diagnosis and treatment of human trichinellosis. Expert Opin. Pharmacother..

[11] Gottstein B., Pozio E., Nöckler K. (2009). Epidemiology, diagnosis, treatment and control of trichinellosis. Clin. Microbiol. Rev..

[12] European Food Safety Authority and European Centre for Disease Prevention and Control (EFSA and ECDC) (2018). The European Union summary report on trends and sources of zoonoses, zoonotic agents and food-borne outbreaks in 2017. EFSA J..

[13] Badagliacca P., Di Sabatino D., Salucci S., Romeo G., Cipriani M., Sulli N., Dall’Acqua F., Ruggieri M., Calistri P., Morelli D. (2016). The role of the wolf in endemic sylvatic *Trichinella britovi* infection in the Abruzzi region of Central Italy. Vet. Parasitol..

[14] Commission Regulation (EC) No 2075/2005 of 5 December 2005 Laying Down Specific Rules on Official Controls for Trichinella in Meat. https://eur-lex.europa.eu/LexUriServ/LexUriServ.do?uri=OJ:L:2005:338:0060:0082:EN:PDF.

[15] Franssen F., Swart A., van der Giessen J., Havelaar A., Takumi K. (2017). Parasite to patient: A quantitative risk model for *Trichinella* spp. pork and wild boar meat. Int. J. Food Microbiol..

[16] Commission Implementing Regulation (EU) No 1375/2015 of 10 August 2015 Laying Down Specific Rules on Official Controls for Trichinella in Meat. https://eur-lex.europa.eu/legal-content/EN/TXT/PDF/?uri=CELEX:32015R1375&from=EN.

[17] Regulation (EC) No 882/2004 of the European Parliament and of the Council of 29 April 2004 on Official Controls Performed to Ensure the Verification of Compliance with Feed and Food Law, Animal Health and Animal Welfare Rules. http://www.cefas.co.uk/media/1550/extract_reg_no_882_2004.pdf.

[18] Zhai P., Wang R., Zhou Y., Hu D., Ii J., Zhou Y. (2019). Enhancing the capabilities of biosafety laboratories through the established accreditation system: Development of the biosafety laboratory accreditation system in China. J. Biosaf. Biosecur..

[19] Regulation (EC) No 765/2008 of the European Parliament and of the Council of 9 July 2008 Setting out the Requirements for Accreditation and Market Surveillance Relating to the Marketing of Products and Repealing Regulation (ECC) No 339/1993. https://eur-lex.europa.eu/LexUriServ/LexUriServ.do?uri=OJ:L:2008:218:0030:0047:EN:PDF.

[20] Ricci U., De Sanzo C., Carboni I., Iozzi S., Nutini A.L., Torricelli F. (2013). Accreditation of a forensic genetics laboratory in Italy. Forensic Sci. Int.: Gen. Suppl. Ser..

[21] Ministry of Health Esame Trichinoscopico Delle Carni di Suini Domestici—Deroga ai Sensi del Regolamento (UE) No. 1375/2015. https://www.anmvioggi.it/images/CIRCOLARE_DEL_MINISTERO_DELLA_SALUTE_copy_copy.pdf.

[22] Ministry of Health Monitoraggio per il Controllo Delle Trichine ai Sensi Dell’articolo 11 del Regolamento (UE) No 1375/2015. http://www.ulss4.veneto.it/web/ulss4/Prevenzione/dfsa/normative/norme_nazionali/note_circolari_minsal/0_pagina_iniziale/all/2016/monit_contr_trichine_Reg_UE_2015_1375.pdf.

[23] Joint ILAC/ISO Communiqué on the Recognition of ISO/IEC 17025 during a Three-Year Transition, 01 November 2017 http://www.esyd.gr/pweb/s/20/files/anakoinoseis/Joint_ILAC-ISO_Com_-_ISO_IEC_17025_transition.pdf.

[24] Marucci G., Tonanzi D., Cherchi S., Galati F., Bella A., Interisano M., Ludovisi A., Amati A., Pozio E. (2016). Proficiency testing to detect *Trichinella* larvae in meat in the European Union. Vet. Parasitol..

[25] International Commission on Trichinellosis Recommendations for Quality Assurance in Digestion Testing Programs for *Trichinella*, ICT Quality Assurance Committee (Appendix 1) Part 3, 2012; pp. 1–9. http://www.trichinellosis.org/uploads/PART_3__final__-_PT_7Feb2012.pdf.

[26] EFSA Panels on Biological Hazards (BIOHAZ), on Contaminants in the Food Chain (CONTAM), and on Animal Health and Welfare (AHAW) (2011). Scientific Opinion on the public health hazards to be covered by inspection of meat (swine). EFSA J..

